# Accuracy of Mandibular Removable Partial Denture Frameworks Fabricated by 3D Printing and Conventional Techniques

**DOI:** 10.3390/ma17133148

**Published:** 2024-06-27

**Authors:** Soonam Kim, Kyung Chul Oh, Jee-Hwan Kim

**Affiliations:** 1 Department of Dentistry, Graduate School, Yonsei University, Seoul 03722, Republic of Korea; snkim2016@gmail.com; 2 Department of Prosthodontics, College of Dentistry, Yonsei University, Seoul 03722, Republic of Korea; kyungabc@yuhs.ac

**Keywords:** accuracy, removable partial denture framework, selective laser melting, superimposition, 3D printing

## Abstract

Herein, we used digital superimposition to evaluate the accuracy of metal frameworks for mandibular removable partial dentures fabricated using three techniques. Thirty master casts of a mandibular dentiform were categorized into three groups (*n* = 10) based on the framework manufacturing method: selective laser melting-based metal three-dimensional (3D) printing (SLM), digital light projection-based resin 3D printing and subsequent casting (RPC), and conventional casting (CON). The master casts were scanned twice, initially after preparation and subsequently after attaching silicone using the frameworks. These scan files were digitally superimposed to measure the silicone thickness. Statistical analysis was conducted using SPSS Statistics (Version 23.0, IBM Corp., Somers, NY, USA). One-way ANOVA and a post hoc Tukey’s multiple comparison tests were performed to determine differences among the three groups (α = 0.05). The RPC group exhibited significantly higher overall and mean internal discrepancies at rest and tissue stops than the SLM and CON groups, which exhibited statistically insignificant differences. Thus, SLM fabrication resulted in comparable accuracy to that achieved by CON, whereas sequentially performing resin 3D printing and casting induced inferior accuracy. However, all frameworks across the three groups were clinically acceptable.

## 1. Introduction

Manufacturing methods for the metal frameworks of removable partial dentures (RPDs) have recently transitioned from conventional lost-wax techniques to computer-aided design (CAD) and computer-aided manufacturing (CAM) techniques, specifically metal three-dimensional (3D) printing. Considering the history of the manufacturing methods of RPD metal frameworks, the transition to the current direct 3D-printed RPD frameworks provides an innovative path. Fauchard first reported the use of metal structures in an RPD in 1728, whereby he used metal labial and lingual bars to connect two carved ivory blocks of the designed RPD [1,2]. In the late 1890s, over 160 years later, the lost-wax casting technique was introduced in dentistry to fabricate metal prostheses, including RPD metal frameworks. However, it comprised complex procedures, the making of a refractory cast, waxing, investing, and casting, thereby requiring a large amount of labor and time [3,4,5]. Moreover, the distortion of the wax pattern on a refractory cast caused inaccuracies in the RPD frameworks, which can hinder the success of the RPD treatment [6]. Despite these drawbacks, conventional casting techniques were used for over a century until CAD/CAM technology was introduced in dentistry in the 1970s, bringing revolutionary changes in the manufacturing methods for all components from inlays to RPD metal frameworks [7,8,9,10,11].

Early CAD/CAM methods involved milling, which is a subtractive technique. However, this technique had several disadvantages, such as the wear of cutting tools, complicated shapes or undercut areas, wastage of cutting chips, long processing time, and shrinkage [12,13]. As such, 3D printing technology has replaced the milling method as it bypasses these limitations. It is an additive manufacturing (AM) technique that produces 3D objects by adding materials in layers, rendering it highly efficient in manufacturing complex-shaped objects [4]. Currently, several 3D printing technologies, such as stereolithography (SLA), digital light projection (DLP), selective laser sintering (SLS), and selective laser melting (SLM), are used depending on the material and energy sources. In this study, SLM and DLP were used [9,10,11,14].

SLA was the first 3D printing technique patented in the 1980s and has been used to manufacture various objects. It involves using ultraviolet lasers to polymerize resin in layer thicknesses ranging from 10 to 100 µm [15,16]. DLP, which was invented by Larry Hornbeck of Texas Instruments in 1987 [17], has a resolution and use range similar to that of SLA. In fact, DLP and SLA are considered to be in the same AM category by the American Section of the International Association for Testing Materials [17]. However, the primary difference between these methods is the light source, that is, the image is formed by an arc lamp or micromirrors, respectively. DLP has a shorter processing time than SLA as the projecting light can cure an entire layer at once [16,18]. The number of micromirrors determines the resolution of the projected image in DLP [17,19]. SLA was first used to print resin sacrificial patterns for RPD frameworks from 3D CAD models in the early 2000s; however, the method required casting [20]. Later, the direct printing of metal RPD frameworks using an SLM 3D printer was achieved, thereby eliminating the complex conventional casting processes, except for finishing and polishing [21,22].

SLM involves melting metal powder using a high-energy laser beam, fusing, and solidifying it in layers according to the CAD information [14,16]. SLM and SLS principles are similar; however, the primary difference between the two methods is the material used [14], that is, SLS and SLM are preferred for ceramics/polymers and metals, respectively. Meanwhile, Suzuki et al. [13] stated that various materials, including metals, could be used in SLS, and the difference between SLS and SLM is the method of manipulating the powder. In particular, SLM involves melting the powder, whereas SLS entails sintering it. Alageel et al. [22] stated that SLM involves the full melting of metal powder, whereas SLS involves its partial melting. In this study, the term SLM implies the metal 3D printing technique.

The use of SLM-fabricated RPD frameworks has increased recently; however, conventional casting techniques remain time- and labor-intensive. To generalize the use of convenient metal 3D-printed frameworks, the advantageous properties of 3D-printed chrome–cobalt (Co–Cr) alloy should be proven, and the accuracy of the frameworks should be comparable to that of conventional cast frameworks. Several studies have reported that SLM-fabricated Co–Cr alloys have more homogeneous microstructures than cast Co–Cr alloys, resulting in enhanced mechanical properties [14,23,24,25,26,27]. For the accuracy of metal 3D-printed RPD frameworks, recent review articles concluded that RPD frameworks fabricated with SLM and conventional casting exhibited similar accuracy within the clinically acceptable range [13,27,28,29]. However, certain studies have noted that although the accuracy values are clinically acceptable, the internal discrepancies of SLM-fabricated RPD frameworks are larger than those of conventionally cast frameworks [30,31,32]. Moreover, the studies on mandibular 3D-printed RPD frameworks are limited. Mandibular RPD frameworks are u-shaped and have a significantly smaller contact area with tissue than maxillary RPD frameworks owing to the absence of a palatal area, where most of the contact with maxillary RPD frameworks is concentrated, which might affect the internal adaptation of the components of RPD frameworks. In addition, despite the various methods employed to investigate the accuracy of RPD frameworks manufactured through CAD/CAM techniques [30,31,32,33,34,35,36,37], comparative studies on the three types of fabrication methods using reliable digital measurements are rare. Previous research predominantly compared metal 3D printing with conventional casting techniques, often relying on point measurements, which have higher contingency compared to area measurements.

In this study, we compared the accuracy of mandibular RPD frameworks fabricated using three different methods through digital analysis with area measurement. The methods evaluated were SLM-based metal 3D printing, DLP-based resin 3D printing followed by casting, and conventional lost-wax casting. The null hypothesis was that there would be no significant differences in the accuracy of the three mandibular RPD metal frameworks.

## 2. Materials and Methods

### 2.1. Master Cast Fabrication and Evaluation of Trueness

A Kennedy Class II Modification 1 mandibular dentiform (YS-RPD; M. Tech, Gimcheon, Republic of Korea) was used in this study. The dentiform was scanned using a tabletop scanner (T500; Medit, Seoul, Republic of Korea) after preparing the rest seats and guiding planes for the left second premolar, right first premolar, and right second molar. The scanning data were saved as a reference file in standard tessellation language (STL). Thirty master casts were fabricated through repeated impressions of the dentiform with vinyl polysiloxane (Aquasil XLV; Dentsply Sirona, Konstanz, Germany) and pouring with a type 4 ultrahard die stone (Snow Rock Gypsum; DK Mungyo Co., Gimhae, Republic of Korea). The master casts were divided into groups of 10 for metal 3D printing (SLM group); 10 for the combination method, resin 3D printing, and casting (RPC group); and the remaining 10 for conventional casting (CON group).

All casts were individually scanned using the same tabletop scanner, and the data were saved as STL files. To prove that the master casts of the three groups did not differ, every STL file of the 30 master casts was individually superimposed over the reference file. The trueness of each master cast was verified using the best-fit alignment of the metrology software (GOM Inspect 2018; Carl Zeiss GOM Metrology GmbH, Braunschweig, Germany).

### 2.2. Fabrication of RPD Frameworks Using Three Methods

**Figure 1:**
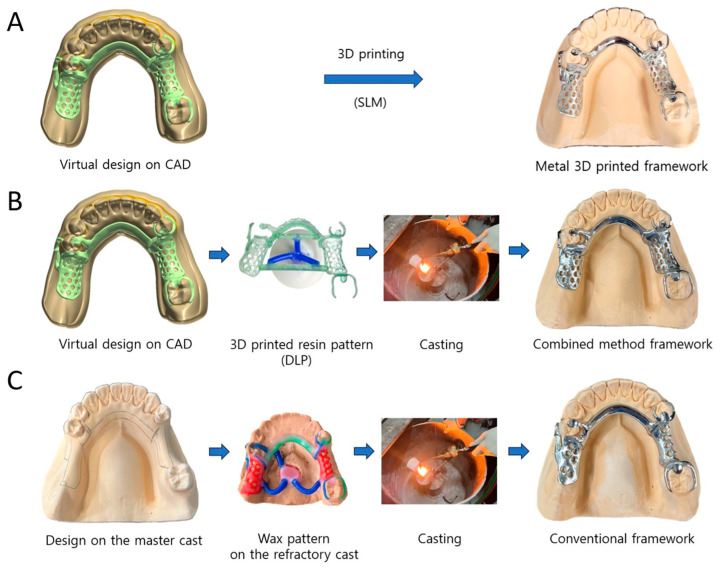
Three manufacturing methods for mandibular RPD metal frameworks: (**A**) SLM-based metal 3D printing (SLM group), (**B**) combined method of DLP-based resin 3D printing and casting (RPC group), and (**C**) conventional lost-wax casting (CON group).

**Figure 2:**
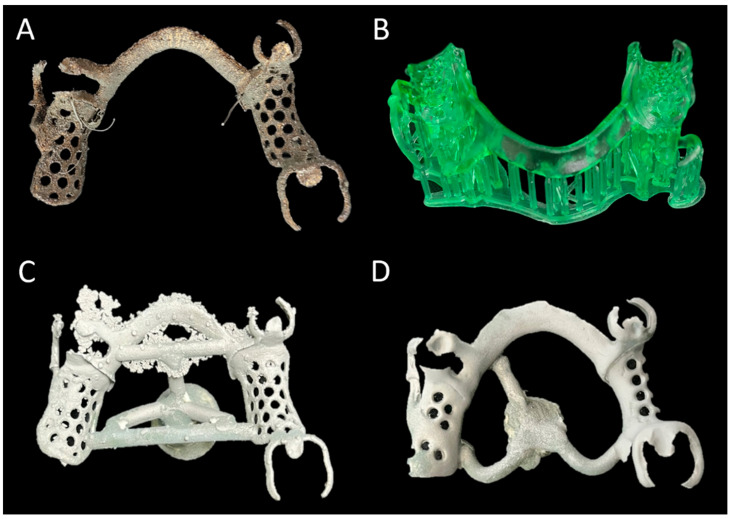
Three kinds of mandibular RPD metal frameworks prior to finishing and 3D-printed resin pattern: (**A**) metal 3D-printed framework, (**B**) 3D-printed resin pattern for RPC group, (**C**) 3D-printed resin-cast framework, and (**D**) conventional cast framework. Note that the shape of (**C**) is identical to (**A**); however, the color of (**C**) is identical to (**D**) due to the use of the same casting alloy.

All 30 RPD frameworks were identically designed to have a lingual bar as a major connector, an I-bar-type clasp on the left second premolar, and basic C clasps on the right first premolar and second molar. The frameworks of the SLM group were designed with CAD software (Dental system 2019; 3Shape A/S, Copenhagen, Denmark) after electronic surveying, and the virtual frameworks were printed out directly using an SLM technology-based metal 3D printer (NCL-M2150X; Nanjing Chamlion Laser Technology Co., Nanjing, China) using Co–Cr alloy powder (ChamTiger; Shinseki International Inc., Seoul, Republic of Korea). The support-attached 3D-printed frameworks were subjected to heat treatment according to the manufacturer’s instructions to release stress and optimize the mechanical properties (Figure 1A). The frameworks of the RPC group were designed using the same CAD software; however, the virtual frameworks were printed out as resin sacrificial patterns using a DLP technology-based resin 3D printer (Pro3D printer SRP1902A; SprintRay Inc., Los Angeles, CA, USA) and 3D-printable resin material (S-plastic cast 2.0; Graphy Inc., Seoul, Republic of Korea). Conventional investing and casting were performed on the 3D-printed castable frameworks (Figure 1B). Finally, for the CON group, the framework design was manually drawn on each master cast, and the refractory casts were fabricated through impressions of the master casts with a reversible hydrocolloid material (Polyflex; Dentsply Sirona, Konstanz, Germany) and pouring (rema Exakt; Dentaurum GmbH, Ispringen, Germany). The wax patterns on the refractory casts were invested with a phosphate-bonded investment material (BC-VEST P-Plus; Bukwang, Busan, Republic of Korea) and cast with the Co–Cr alloy (Biosil F; Degudent, Hanau, Germany) (Figure 1C). Tissue stops of the CON group were made in the same location as in the SLM and RPC groups. The three kinds of mandibular RPD metal frameworks prior to finishing and polishing and the 3D-printed resin pattern for the RPC group are presented in Figure 2. Finishing and polishing processes were performed on 30 RPD frameworks to be adapted to the corresponding master casts. The design procedures were implemented by an experienced prosthodontist, and all laboratory procedures were performed by an experienced board-certified dental laboratory technician.

### 2.3. Scanning of the Master Casts with Silicone Material

Each master cast in the three groups was scanned with the tabletop scanner to obtain the STL file of the cast only, which was essential for the superimposition in the subsequent measurement step (Section 2.4). Each RPD framework was fitted onto the corresponding master cast using silicone. Before fitting, the intaglio surface of the framework was coated with a thin layer of petroleum jelly (Vaseline, Unilever, Greenwich, CT, USA) to prevent the silicone from attaching to the framework. Additionally, a thin adhesive liquid (3M ESPE Polyether Adhesive, 3M ESPE AG, Bayern, Germany) was applied to the measuring areas of the cast to prevent detachment of the silicone during framework removal. Mixed vinyl polyether silicone (Fit Checker Advanced; GC Corp., Tokyo, Japan) was applied to the intaglio surface of the rests, tissue stops, and lingual bars. The framework was immediately fitted onto the corresponding cast and carefully removed after the silicone hardened, leaving the silicone intact on the casts. If any gap was detected between the silicone and the measuring area of the cast, the process was repeated after cleaning the cast. Each master cast with silicone was rescanned using the tabletop scanner. All scanning procedures were performed without a powder coating.

### 2.4. Measurement of the Internal Discrepancies of the RPD Frameworks

**Figure 3:**
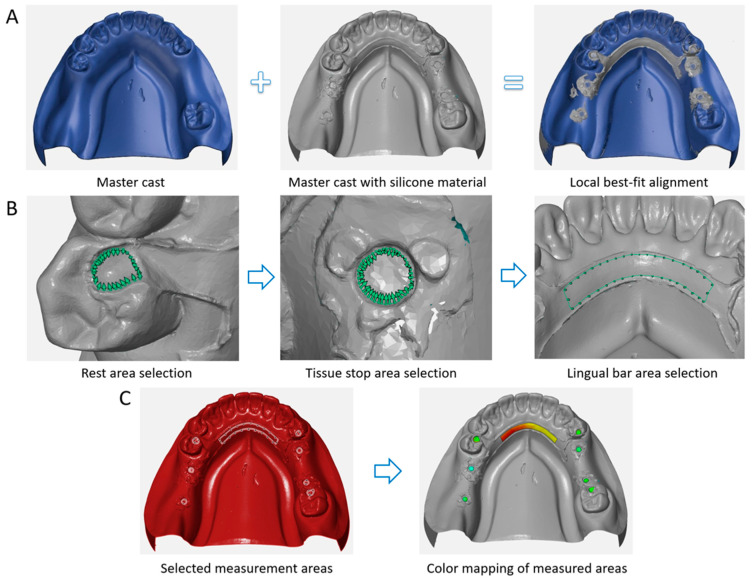
Screenshots of the measurement steps with the 3D metrology software. The thickness of the imprinted silicone material representing the internal discrepancies was measured by superimposing two STL files: the cast only and the silicone-attached cast. (**A**) Superimposition of the two STL files by local best-fit alignment function of the metrology software, (**B**) manual selection of each measurement area, and (**C**) selected border lines at eight measurement areas (three rests, four tissue stops, and one lingual bar area) and color mapping of the areas (green represents good fit, yellow to red represents positive error, blue represents negative error).

The measurement steps using the 3D metrology software are presented in Figure 3. The two types of STL files, namely that of the master cast only and the silicone-attached master cast, were superimposed using a local best-fit alignment function of the metrology software (GOM Inspect 2018, Carl Zeiss GOM Metrology GmbH, Braunschweig, Germany) to measure the thickness of the silicone, representing the internal discrepancy of the framework (Figure 3A). The mean deviation values in the eight areas of the RPD framework were measured: three rests (35R, 44R, and 47R), four tissue stops (36T, 37T, 45T, and 46T), and a lingual bar. The borderlines of the measuring areas were manually created in each master cast following a certain standard to ensure designation consistency. Each borderline was chosen to be slightly away from the edge, and the perpendicular borders of lingual bars were selected at the distal sides of the canines (Figure 3B). Each selected patch was inspected using the “surface comparison on actual” function of the metrology software and visualized using color mapping and tabulating the mean and maximum deviation values (Figure 3C). 

The internal discrepancies at the rests, tissue stops, and lingual bars (IDR, IDT, and IDL, respectively) were measured. In addition, the overall internal discrepancy (IDO), comprising the average values of the IDR and IDT, was calculated.

### 2.5. Statistical Analysis

The sample size of 10 per group was determined based on the statistical significance level set at α = 0.05 and power (1 − β) of 0.8 with an effect size of 0.6. The Shapiro–Wilk test was performed to examine the normality and all data followed normal distribution (*p* > 0.05). One-way analysis of variance and a post hoc Tukey’s multiple comparison test were performed to determine differences between the three groups (α = 0.05). The data were analyzed using statistical software (SPSS Statistics version 23.0, IBM Corp., Somers, NY, USA), and the analysis graphs were created using the graphing software (GraphPad Prism 10, Boston, MA, USA).

## 3. Results

**Figure 4:**
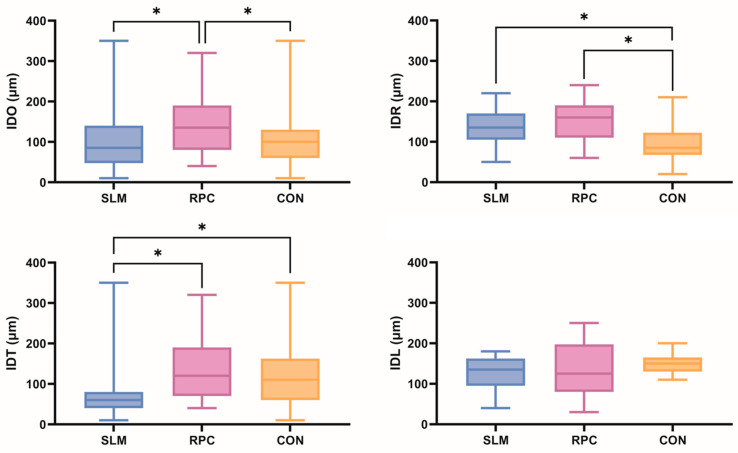
Comparisons of the overall internal discrepancies (IDOs) and internal discrepancies at rests, tissue stops, and lingual bars (IDR, IDT, and IDL, respectively) of mandibular RPD metal frameworks fabricated using three methods (SLM: selective laser melting-based metal 3D printing, RPC: DLP-based resin 3D printing and subsequent casting, CON: conventional lost-wax casting). The asterisks indicate statistically significant differences among the three groups (*p* < 0.05).

The overall internal discrepancies (IDOs) and internal discrepancies at rests, tissue stops, and lingual bars (IDR, IDT, and IDL, respectively) of mandibular RPD metal frameworks fabricated using three methods are presented in Figure 4. The RPC group shows significantly higher IDOs than the SLM and CON groups (*p* = 0.001 and 0.019, respectively), whereas the IDOs of the SLM and CON groups do not differ significantly (*p* = 0.633). The CON group has the lowest mean IDR (*p* = 0.010, *p* < 0.001), whereas the SLM group has the lowest IDT (*p* = 0.001, *p* = 0.025). The RPC group, combined with resin 3D printing and casting, shows the highest IDRs and IDTs with significant differences. For the IDLs, no significant differences are noted among the three groups. The mean IDRs, IDTs, and IDLs of the mandibular RPD frameworks in the three groups are presented in Table 1.

## 4. Discussion

The null hypothesis was rejected owing to significant differences in the accuracy of the mandibular RPD metal frameworks fabricated using the three manufacturing methods. There were no significant differences between the IDOs of the SLM and CON groups, whereas the RPC group showed a higher IDO than the other two groups, which can be ascribed to the higher error tendency of the RPD frameworks of the RPC group. Although the RPC group eliminated the steps of fabricating a refractory cast and waxing, which could result in inaccuracies owing to the physical properties of wax [6], it was implicated in the errors from the steps of 3D printing of castable resin patterns and in those from the steps of conventional investing and casting. According to Revilla-Leon and Ozcan [17], discrepancies can be incorporated into each step of a digital dental workflow. The 3D printer parameters, the material used (which has its optimal activation range of wavelength), power, and exposition time for AM on the 3D printers can affect the accuracy of the printed objects [17].

The accuracy of the rests and tissue stops, which are structural components of the RPD frameworks that directly contact the tooth or tissue, differed significantly among the three groups. The CON group showed the highest accuracy for the rests, and the SLM group had the highest accuracy for tissue stops. The RPC group had the lowest accuracy for both components. In 2020, Tasaka et al. [36] reported that the accuracy of mandibular RPD metal frameworks differed depending on the structural component comparing SLS technology-based 3D printing and 3D-printed resin pattern casting, which is consistent with the results of this study. In 2019, Bajunaid et al. [37] compared the accuracy of mandibular RPD frameworks fabricated by SLM technology-based 3D printing and conventional casting through measuring four rest zones with a digital microscope. The zones with the highest fit and accuracy among the two groups differed, which is also consistent with this study.

As this study was conducted on the mandibular RPD frameworks, it was also compared with previous studies on the maxillary RPD frameworks. Oh et al. [35] compared the accuracy of maxillary RPD metal frameworks under similar conditions with this study, concluding that there were no significant differences among the three groups of SLM, RPC, and CON; however, the IDOs (226.99–365.30 μm) were all higher than those of this study (101.70–143.70 µm). The mean IDR of the three groups in this study was also lower by approximately 110 μm than that of the study of Oh et al. [35]. This difference could be ascribed to different factors. First, the palatal contact area was not included in the mandibular RPD frameworks, which can reduce the interferences before the contact of rests and rest seats. In contrast, the maxillary RPD frameworks could have early interferences due to the palatal contact of a major connector. Second, the measurement criteria differed. “Point” measurement was employed in the previous study, whereas “area” measurement was employed in this study. The “area” measurement can reduce the contingency of manual designation more than the “point” measurement since it includes countless points within the designated border lines. Lastly, the use of equipment and metrology software from different manufacturing companies could affect the difference. Chen et al. [38] evaluated the adaptation of maxillary RPD metal frameworks fabricated by the SLM technique with four types of partially edentulous resin models. They reported that SLM-fabricated RPD frameworks had acceptable accuracies; however, among the frameworks with a large span and more retainers and clasps, the conventional casting technique exhibited slightly better fit and accuracy. Moreover, Soltanzadeh et al. [30] evaluated the accuracy and fit of maxillary RPD frameworks fabricated by conventional casting and 3D printing techniques with stone and 3D-printed resin models. Both methods revealed clinically acceptable adaptation (50–311 µm), but the conventional casting groups exhibited better overall fit and higher accuracy. The poorest fit was observed at the anterior palatal straps fabricated using the 3D printing technique.

Studies on 3D-printed RPD metal frameworks regardless of maxilla and mandible have previously been conducted. Tregerman et al. [16] compared the clinical fit of RPD metal frameworks fabricated by three workflows—conventional casting pathway, SLM 3D printing with extraoral scanning of the stone cast, and SLM 3D printing with intraoral scanning—and concluded that the completely digital workflow had the lowest misfit. Almufleh et al. [39] compared patient satisfaction with RPDs using frameworks fabricated by conventional casting and SLS 3D printing, revealing that higher satisfaction was achieved with the RPDs obtained using SLS 3D-printed frameworks. Peng et al. [40] compared the trueness of RPD metal frameworks fabricated by SLM and 3D-printed resin-casting. The frameworks fabricated by SLM 3D printing exhibited higher trueness than those by the combined method. Summarizing the results of recent studies, SLM 3D printing and conventional casting techniques demonstrated similar accuracies for fabricating RPD metal frameworks within a clinically acceptable range, as evidenced in this study.

For the internal gap between a rest and a rest seat, the mean distance per rest was reported as 69–387 µm [41] and 193–203 µm [42]. Lee et al. [34] studied the accuracy of RPD frameworks fabricated through a combined method. The mean IDR of 249.27 ± 134.84 µm, which was higher than previously reported values on casted RPD frameworks, was obtained. Oh et al. [35] obtained IDRs in the range of 211.91 ± 16.84 to 259.26 ± 45.41 µm without significant differences among the three manufacturing methods. Souza Curinga et al. [27] achieved the IDR range of 20–279 µm for conventional cast frameworks and 30–272 µm for 3D-printed frameworks, which did not indicate a significant difference. The IDRs in the aforementioned studies are in the range of 20–387 µm, whereas those in this study were lower, indicating clinically acceptable values.

The internal discrepancies of lingual bars, which have not been previously investigated, were also measured in this study, whereby no significant differences were noted among the three groups. The default value of 200 µm was set for lingual relief in CAD subtracted from the IDLs of the SLM and RPC groups because wax relief under a lingual bar was not performed in the CON group owing to the absence of undercuts. We also tried to identify specific tendencies depending on the locational factors among three groups, such as the tooth-borne area versus tooth and mucosa-borne area; however, it was difficult to observe particular trends. The table and figures of the additional regional analysis are included in the Supplementary Materials (Supplemental Table S1, Figures S1 and S2).

Nonetheless, this study had certain limitations. First, this work was an in vitro study with different conditions than that with an actual patient’s oral mucosa. The edentulous area of a patient is covered with elastic soft tissue and saliva, whereas the master cast is not. Thus, there might be differences compared to results obtained from in vivo studies. Second, finishing and polishing can have various influences on the results. However, the complete adaptation of the RPD framework on the cast is difficult to achieve without finishing and polishing, and an actual RPD framework is fitted into the patient’s oral cavity after complete polishing. Therefore, the measurement of polished frameworks was considered inevitable and appropriate. However, all 30 frameworks were finished and polished by a single experienced board-certified laboratory technician to minimize the undesirable effect on the result of this study. If the processes that need to be applied equally to all samples, such as finishing and polishing, were carried out by different technicians, it would be difficult to ensure accurate comparisons among the groups since this study was based on an experiment requiring group comparison under the same conditions. Lastly, the manual designation of the border lines of the measurement areas can affect the measurement values finely. Owing to the nature of digital measurement in the metrology program, the value changes finely each time it is measured depending on the selected location. Therefore, we conducted measurements multiple times with careful selection of the border lines.

However, to date, metal 3D printing using SLM technology is the most convenient method for manufacturing RPD frameworks, which can reduce the time and labor required for conventional laboratory processes. To produce more accurate RPD frameworks than conventional cast frameworks, further studies using various metal 3D printers and software programs are essential. Further, more in vivo studies on the accuracy, fitness, and longevity of metal 3D-printed RPD frameworks are needed until the use of metal 3D-printed RPD frameworks becomes generalized.

Overall, the following conclusions were drawn in this study: (1) SLM-fabricated RPD frameworks exhibited an overall accuracy similar to that of conventional cast RPD frameworks; and (2) a combined method of resin 3D printing and casting demonstrated inferior accuracy. However, all frameworks in the three groups were clinically acceptable.

## Figures and Tables

**Table 1:** Internal discrepancies (µm) of the mandibular RPD metal frameworks fabricated using three methods.

Internal Gap/Group	SLM	RPC	CON
IDO	101.7 ± 68.41 ^a^ (81.48–121.95)	143.7 ± 67.29 ^b^ (123.43–163.99)	112.3 ± 70.09 ^a^ (88.83–135.74)
IDR	133.0 ± 43.87 ^a^ (120.92–145.08)	149.0 ± 50.33 ^a^ (127.09–170.91)	96.3 ± 48.74 ^b^ (78.02–114.65)
IDT	78.2 ± 74.38 ^a^ (49.23–107.27)	139.8 ± 78.03 ^b^ (114.02–165.48)	124.3 ± 81.14 ^b^ (88.66–159.84)
IDL	127.0 ± 44.48 ^a^ (95.18–158.82)	135.0 ± 72.91 ^a^ (82.84–187.16)	150.0 ± 25.81 ^a^ (131.53–168.47)

The data are expressed as mean ± standard deviation and confidence intervals. IDO, overall internal discrepancy; IDR, internal discrepancy of rests; IDT, internal discrepancy of tissue stops; IDL, internal discrepancy of the lingual bar; SLM group, metal 3D-printed frameworks; RPC group, 3D-printed resin-cast frameworks; CON group, conventional cast frameworks. The default value of 200 µm was subtracted from IDL in SLM and RPC groups. The different letters indicate statistically significant differences among the three groups (*p* < 0.05).

## Data Availability

The data presented in this study are available on request from the corresponding author.

## Supplementary Materials

The following supporting information can be downloaded at https://www.mdpi.com/article/10.3390/ma17133148/s1, Table S1: The internal discrepancies (µm) at three rests and four tissue stops in mandibular RPD metal frameworks of three groups; Figure S1: Comparisons of the internal discrepancies at the rests of #35, #44, #47 in mandibular RPD metal frameworks fabricated using three methods (SLM: selective laser melting-based metal 3D printing, RPC: DLP-based resin 3D printing and subsequent casting, CON: conventional lost-wax casting); Figure S2: Comparisons of the internal discrepancies at the tissue stops of #36, #37, #45, #46 in mandibular RPD metal frameworks fabricated using three methods (SLM: selective laser melting-based metal 3D printing, RPC: DLP-based resin 3D printing and subsequent casting, CON: conventional lost-wax casting).
