# Supplementary material for: Accuracy of Mandibular Removable Partial Denture Frameworks Fabricated by 3D Printing and Conventional Techniques
Source: Materials (Basel). 2024 Jun 27;17(13):3148. doi: 10.3390/ma17133148 (PMC11242778; doi:10.3390/ma17133148)
Supplement: Supplementary file 1 [file materials-17-03148-s001.zip › materials-3052828-supplementary (1).pdf]

Supplemental Table S1. The internal discrepancies (μm) at three rests and four tissue stops in mandibular RPD metal frameworks of three groups. The data are expressed as mean ± standard deviations. SLM group, metal 3D-printed frameworks; RPC group, 3D-printed resin-cast frameworks; CON group, conventional cast frameworks. Different lowercase letters in the same row indicate statistically significant differences among the groups.

Internal gap(μm) / Group	SLM	RPC	CON
**#35 rest**	122 ± 38.23[a]	183 ± 40.83[b]	129 ± 57.62[a]
**#44 rest**	126 ± 42.47[a]	132 ± 46.61[a]	65 ± 32.40[b]
**#47 rest**	151 ± 48.86[a]	132 ± 48.94[ab]	95 ± 31.71[b]
**#36 tissue stop**	59 ± 35.73[a]	87 ± 38.60[ab]	136 ± 50.54[b]
**#37 tissue stop**	104 ± 50.68[a]	109 ± 65.02[a]	69 ± 36.04[b]
**#45 tissue stop**	51 ± 28.06[a]	168 ± 55.39[b]	111 ± 58.47[ab]
**#46 tissue stop**	99 ± 45.54[a]	195 ± 61.39[b]	181 ± 60.33[b]

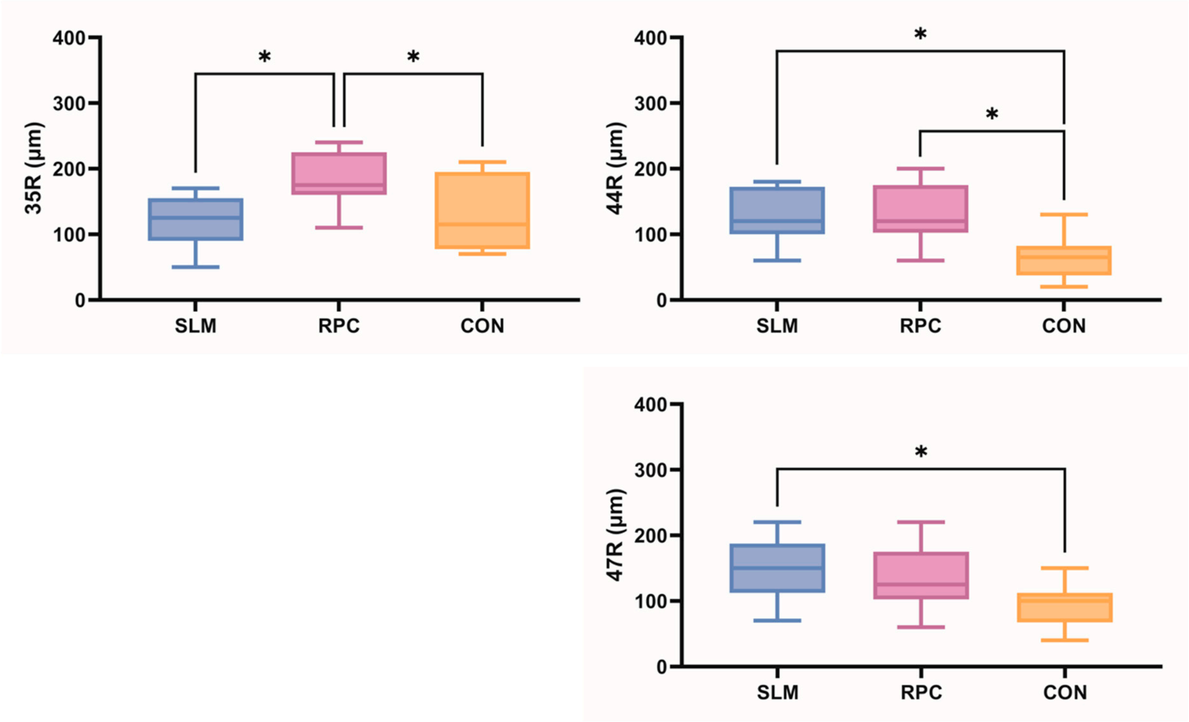

Supplemental Figure S1. Comparisons of the internal discrepancies at the rests of #35, #44, #47 in mandibular RPD metal frameworks fabricated using three methods (SLM: selective laser melting-based metal 3D printing, RPC: DLP-based resin 3D printing and subsequent casting, CON: conventional lost-wax casting). The asterisks indicate statistically significant differences among the three groups.

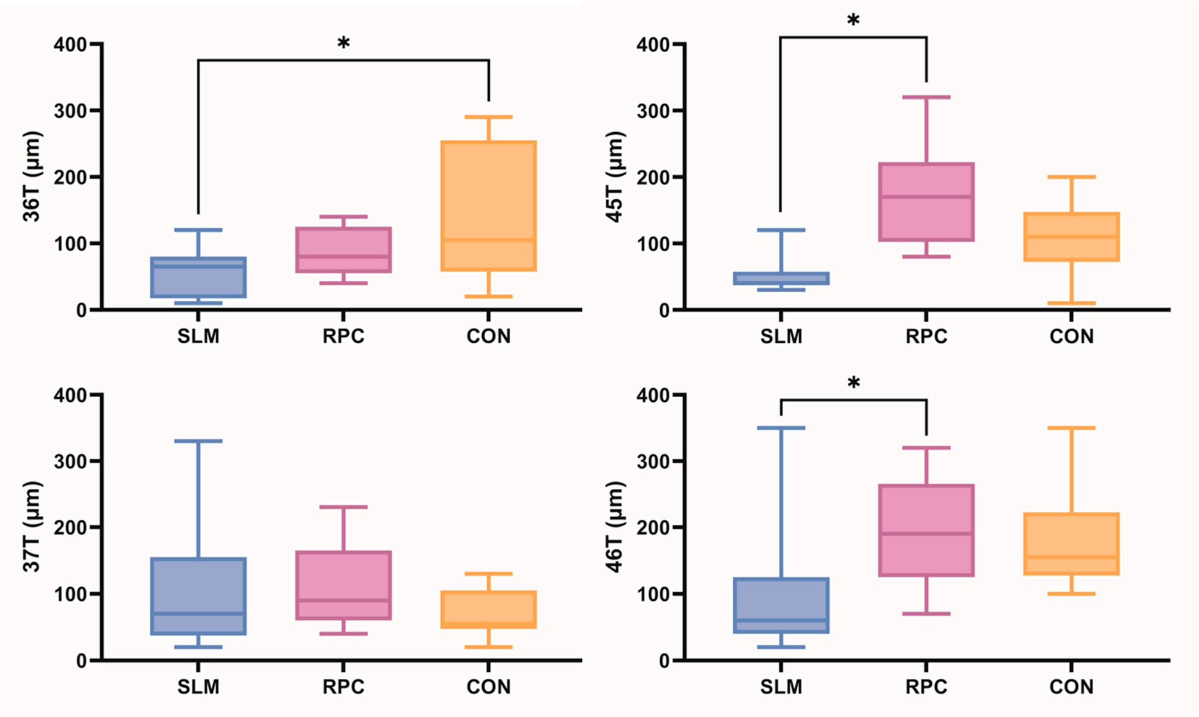

Supplemental Figure S2. Comparisons of the internal discrepancies at the tissue stops of #36, #37, #45, #46 in mandibular RPD metal frameworks fabricated using three methods (SLM: selective laser melting-based metal 3D printing, RPC: DLP-based resin 3D printing and subsequent casting, CON: conventional lost-wax casting). The asterisks indicate statistically significant differences among the three groups.
